# Predictive Tool for Tunnelled Central Venous Catheter Dysfunction in Haemodialysis

**DOI:** 10.3390/jcm14165647

**Published:** 2025-08-09

**Authors:** Verónica Gimeno-Hernán, Jose Antonio Herrero Calvo, Juan Vicente Beneit Montesinos, David Hernán Gascueña, Irene Serrano García, Ismael Ortuño-Soriano

**Affiliations:** 1 Nursing Department, Faculty of Nursing, Physiotherapy and Podology, Universidad Complutense de Madrid, 28040 Madrid, Spain; jvbeneit@enf.ucm.es (J.V.B.M.); iortunos@ucm.es (I.O.-S.); 2 Clinical Pharmacology Department, Instituto de Investigación Sanitaria del Hospital Clínico San Carlos (IdISSC), 28040 Madrid, Spain; 3 Department of Nephrology, Hospital Clínico San Carlos, 28040 Madrid, Spain; jaherrero2@telefonica.net; 4 Nursing Department, Fundación Renal Española, 28040 Madrid, Spain; dhernan@fundacionrenal.es; 5 Methodological Support Unit for Research, Instituto de Investigación Sanitaria del Hospital Clínico San Carlos (IdISSC), 28040 Madrid, Spain; iserrag01@gmail.com; 6 Health Care Research Group, Instituto de Investigación Sanitaria del Hospital Clínico San Carlos (IdISSC), 28040 Madrid, Spain

**Keywords:** chronic kidney disease, dysfunction, haemodialysis, tunnelled central venous catheter, predictive model

## Abstract

**Introduction:** Tunnelled central venous catheters are increasingly used for vascular access in patients undergoing haemodialysis for chronic kidney disease. However, catheter dysfunction is a frequent and clinically relevant complication, impairing treatment efficacy and increasing morbidity. This study aimed to develop and internally validate predictive models for catheter dysfunction using routinely collected haemodialysis session data, with the goal of facilitating early detection and proactive clinical decision-making. **Methods:** We conducted a diagnostic, retrospective, cross-sectional, and analytical study based on 60,230 HD sessions recorded in 2021 across dialysis centres in Spain. A total of 743 patients with functioning catheter were included. Clinical, technical, and haemodynamic variables were analysed to identify those associated with catheter dysfunction in the subsequent session. Five logistic regression models were built; the dataset was split into training (two-thirds) and internal validation (one-third) cohorts. Model performance was evaluated using the area under the ROC curve (AUC) and the Hosmer–Lemeshow test. **Results:** Significant predictors included venous pressure, effective blood flow, catheter location, convective techniques, and line reversal. The bootstrapping model, selected for internal validation due to its parsimony and performance, achieved an AUC of 0.844 (95% CI: 0.824–0.863), with a sensitivity of 81.6% and a specificity of 70.9% at a 0.019 threshold. **Conclusions:** The bootstrapping-based predictive model is a valuable clinical tool for anticipating catheter dysfunction using routine haemodialysis data. Its implementation may enable earlier intervention, reduce reliance on reactive treatments, and enhance vascular access management in haemodialysis patients.

## 1. Introduction

Chronic kidney disease (CKD) is one of the leading causes of morbidity and mortality worldwide, placing an increasing burden on healthcare systems. For patients in the terminal stage, haemodialysis (HD) remains an essential therapeutic modality, and in many of these patients, the tunnelled central venous catheter (TCVC) serves as the preferred form of vascular access, whether used temporarily or permanently. However, the use of these devices is not without complications, with catheter dysfunction among the most frequent and clinically as well as economically significant issues [1,2].

It is estimated that a substantial proportion of patients with a TCVC will experience dysfunction episodes, defined by reduced blood flow compromising dialysis efficacy, specifically, flow rates below 300 mL/min, according to the National Kidney Foundation criteria [1]. This dysfunction is associated with an increased risk of infections, thrombosis, recurrent hospitalisations, higher healthcare resource use, and reduced patient quality of life [3,4,5,6]. Despite this, the management of the complication has largely been reactive, focusing on therapeutic interventions after the clinical event, often resulting in treatment delays and increased complications [7,8].

In recent years, there has been growing interest in implementing preventive and predictive strategies in nephrology. Early identification of dysfunction risk would enable optimised vascular access management, improved health outcomes, and reduced economic burden [9]. However, there remains a lack of simple, effective, evidence-based tools that can anticipate TCVC dysfunction with adequate precision and real-world applicability [10,11].

Although some studies have developed predictive models for vascular access survival, by incorporating clinical, demographic, and technical variables [12,13,14], many of these have limitations, such as excessive complexity, dependence on non-real-time data, limited external validity, and poor integration with electronic health records systems. Additionally, there remains a notable gap in developing models focused specifically on catheter dysfunction as a preventable and predictable clinical event [15].

Considering the unmet need, there is a timely necessity to conduct observational and analytical studies aimed at identifying factors associated with TCVC dysfunction in haemodialysis patients. These studies would support the development of validated, user-friendly predictive models for everyday clinical practice. Such models could enable early interventions to prevent progression to serious complications, avoiding treatment interruptions, unnecessary hospitalisations, and invasive procedures such as catheter replacement [16,17].

Incorporating predictive tools into routine care would offer benefits at multiple levels: clinically, by supporting patient-specific monitoring; organisationally, by optimising resource use and reducing costs; and at the policy level, by promoting standardised, evidence-based vascular access protocols [2,12].

Finally, the aim of this study is to develop and validate a predictive tool for TCVC dysfunction in haemodialysis patients. This will be achieved through observational and analytical evaluation of clinical and demographic variables, supporting preventive decision-making in routine care.

## 2. Methods

**Study design**: An analytical, observational, cross-sectional study with a diagnostic approach was conducted. This design was selected based on the nature of data collection, which involved historical records from haemodialysis sessions, and the temporal relationship between predictor variables and the occurrence or absence of catheter dysfunction in the subsequent session. The study was structured into two distinct phases: model development and training in the first, and internal validation in the second.

**Study setting**: The study was conducted within the haemodialysis services operated by the Fundación Renal, a non-profit organisation managing multiple dialysis centres across Spain.

**Study population:** The study population comprised patients diagnosed with CKD stage 5, prevalent, incident, and fitted with a TCVC, who received care at the specified haemodialysis centres. Inclusion criteria were patients aged over 18 years, enrolled in the Fundación Renal chronic haemodialysis programme, and carrying a TCVC during the data collection period. Patients admitted to hospitals outside the participating centres were excluded due to the inability to ensure traceability and exclusive use of the catheter during these periods.

**Sample size and sampling procedure**: A convenience, non-probabilistic sampling method was used to include the maximum possible number of eligible patients, aiming to maximise model stability and generalisability. All haemodialysis sessions recorded between 1 January and 31 December 2021 at the participating centres were analysed, resulting in a total of 60,230 evaluated sessions.

For model creation and validation, the data were randomly stratified into two subsets using EPIDAT^®^. Two-thirds of the data formed the training set, and the remaining third was used for internal validation. Both subsets preserved the original proportions of events (catheter dysfunction) and non-events, ensuring representativeness and statistical balance for robust model evaluation. The large sample size was crucial for achieving reliable estimations and minimising bias in predictive analysis.

**Study variables:** The primary outcome variable was the occurrence of TCVC dysfunction during the subsequent haemodialysis session. Dysfunction was defined as the inability to maintain adequate blood flow (<300 mL/min) during the first 60 min of treatment, despite corrective measures, in accordance with the National Kidney Foundation guidelines. This included sessions where this limitation was observed for the first time, having achieved ≥300 mL/min in previous treatments. Additionally, dysfunction was defined by the presence of abnormal arterial and/or venous pressures, also based on the National Kidney Foundation guidelines, with arterial pressure values exceeding −250 mmHg or venous pressure values above 250 mmHg. This outcome was treated as a dichotomous variable (dysfunction: yes/no).

Secondary variables were grouped as either dynamic or static. Dynamic variables, which could change between sessions, included the type and timing of dysfunction (arterial or venous), blood flow and pressure at different session points, urea clearance (Kt), volume of blood dialysed, treatment duration, dialysis technique, catheter sealing type, line positioning, dialyser type, and dialysate flow rate. These were classified as qualitative or quantitative, depending on their nature.

Static variables referred to constant patient or access characteristics, including age, sex, body surface area, comorbidities (diabetes, hypertension, or cardiovascular disease), history of SARS-CoV-2 infection, catheter location, number of vascular accesses and previous transplants, anticoagulant or antiplatelet therapy, and CKD aetiology. These were coded as dichotomous, nominal, or continuous as appropriate.

All independent variables were recorded at the end of each haemodialysis session and were included in the multivariate analysis used to develop the predictive model.

This methodology enabled the detailed analysis of TCVC dysfunction in haemodialysis, its effect on treatment efficacy, the identification of risk factors, and strategies to optimise vascular access.

**Data collection:** Strategies to ensure data quality included the careful selection of variables, anonymisation via unique patient identification codes, and the verification of coherence through random sampling and cross-checking against original records. Extracted data were exported in Excel^®^ format as the basis for statistical analysis.

To facilitate efficient data management and processing, Python^®^ was used for cleaning, organisation, and analysis. Dynamic variables linked to haemodialysis sessions were downloaded by centre to minimise system load. The database included catheter line status, arterial and venous pressure values during the session, final Kt as a dialysis efficacy marker, catheter sealing type, and dialyser type, all of which were associated with TCVC dysfunction. Data coherence was further ensured by random checks against original records. This comprehensive data processing provided the foundation for identifying dysfunction risk factors.

**Statistical analysis**: Data analysis comprised two stages: an initial descriptive analysis followed by a predictive analytical phase. Absolute and relative frequencies were calculated for qualitative variables, while quantitative variables were summarised using means and standard deviations (if normally distributed) or medians and interquartile ranges (if not).

Univariate analysis explored associations between each independent variable and catheter dysfunction in the subsequent session. Chi-square or Fisher’s exact tests were used for qualitative variables, and Student’s *t*-test or the Mann–Whitney U-test for quantitative variables, depending on normality.

The complete dataset of 60,230 sessions was randomly stratified into training (66%) and internal validation (33%) subsets. Variables that were statistically significant (*p* < 0.05) in univariate analysis were considered for multivariable modelling.

Five logistic regression approaches were compared: a full model using all significant variables, forward and backward stepwise models, Lasso regression, and a bootstrapping model (200 iterations). Nine variables were ultimately selected. Model performance was compared using AIC, BIC, and the Hosmer–Lemeshow test.

The best-performing models—forward, backward, and bootstrapping—underwent internal validation. Predictive performance was assessed via area under the ROC curve (AUC), with optimal cut-offs selected to balance sensitivity and specificity. The final model was implemented in Excel^®^, providing individual dysfunction probabilities.

All analyses used a significance threshold of *p* < 0.05 and 95% confidence intervals. Processing was performed using SPSS^®^ v26 and STATA^®^.

**Ethical considerations**: This study adhered to international ethical guidelines for research involving human subjects, including the Declaration of Helsinki and the General Data Protection Regulation (EU 2016/679). Ethical approval was granted by the Clinical Research Ethics Committee of Hospital Clínico San Carlos (reference: 22/398-E_Tesis; favourable opinion issued on 11 July 2022).

Informed consent was obtained from all individual participants included in the study.

All personal data were processed with strict confidentiality under Organic Law 3/2018. Anonymisation was achieved through coding, with only the principal investigator able to access the key linking codes to identifiable information.

## 3. Results

A descriptive analysis of the variables was conducted to characterise the study population. During the analysis period, a total of 743 patients (473 women and 270 men) were included, of whom 49.4% were diabetic and 84.5% hypertensive.

The mean age of patients was 68 years (range: 20–97 years), with an average of 1.17 catheters per year and a mean haemodialysis (HD) session duration of 3.7 h. Regarding treatment length, 50% of patients had been receiving dialysis for over 20 months. Most patients with tunnelled central venous catheters (TCVCs) underwent dialysis three times per week, representing a notable deviation from other regimens.

### 3.1. Model Training

A univariate analysis was performed to examine the relationship between each independent variable and subsequent HD session dysfunction. Each HD session was analysed independently, regardless of patient identity, initiating the first phase of model development.

The training dataset was used in this development process. Predictors were selected for model fitting based on the strength of their association with the primary outcome. Variables meeting a significance threshold of *p* < 0.05 were included, allowing their relationship with dysfunction risk to be examined and ensuring rigour in variable selection. The complete model was built using multivariate logistic regression with all statistically significant variables from univariate analysis. The Hosmer–Lemeshow test produced a value of 0.0231 (Table 1), indicating some discrepancies between observed and expected values and suggesting calibration limitations.

**Figure 1:**
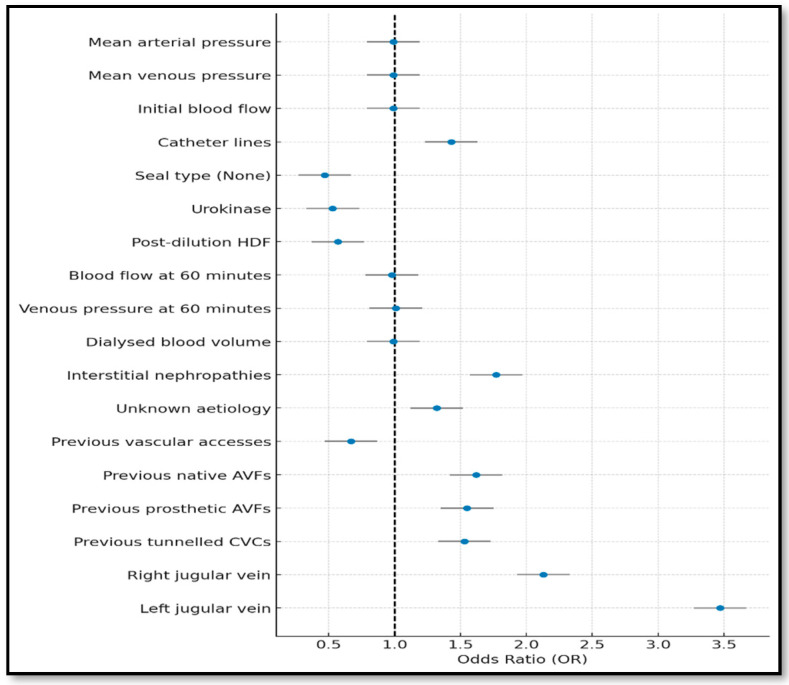
Forest plot of the complete model (variables with *p*-value < 0.05).

Key results are illustrated in Figure 1. Notably, higher mean arterial pressure, venous pressure, initial blood flow, 60 min blood flow, and volume of cleared blood were associated with a progressive 1–2% decrease in dysfunction risk.

Conversely, increased initial venous pressure and venous pressure at 60 min were associated with elevated risk, as was catheter line inversion, which raised the dysfunction likelihood by 43%.

Post-dilutional haemodiafiltration (HDF) showed a protective effect (43% risk reduction), as did certain catheter sealing types, including urokinase and the absence of sealing, both linked to reduced dysfunction risk.

Interstitial nephropathies and unknown aetiologies were strongly associated with dysfunction. Previous vascular accesses lowered the risk, while a prior arteriovenous fistula, native or prosthetic, significantly increased it.

Finally, left jugular vein placement [OR = 3.47; 95%IC (2.27–5.28)] and right jugular vein placement [OR = 2.13; 95%IC (1.35–3.36)] were the anatomical sites most strongly associated with dysfunction.

### 3.2. Stepwise Model (Forward and Backward)

**Figure 2:**
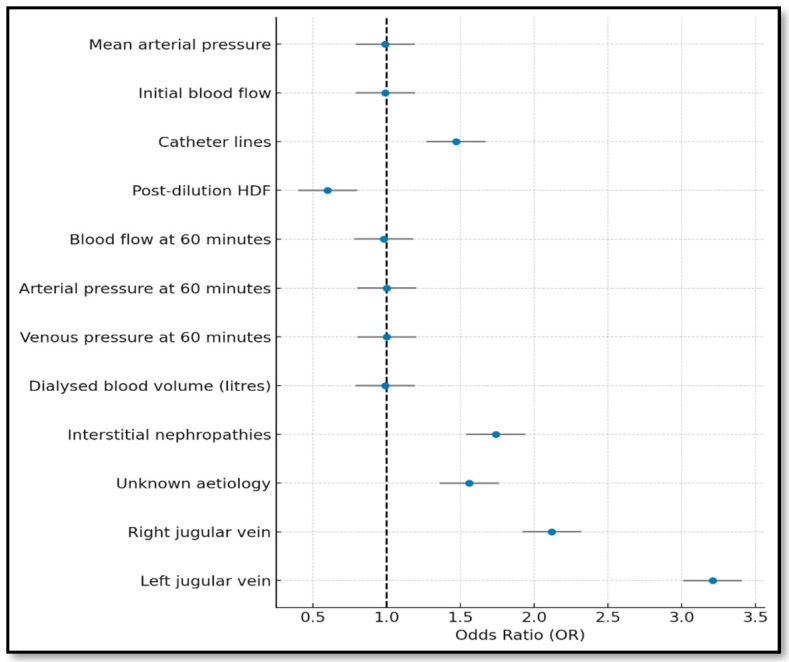
Forest plot of the forward stepwise model (variables with *p*-value < 0.05).

**Figure 3:**
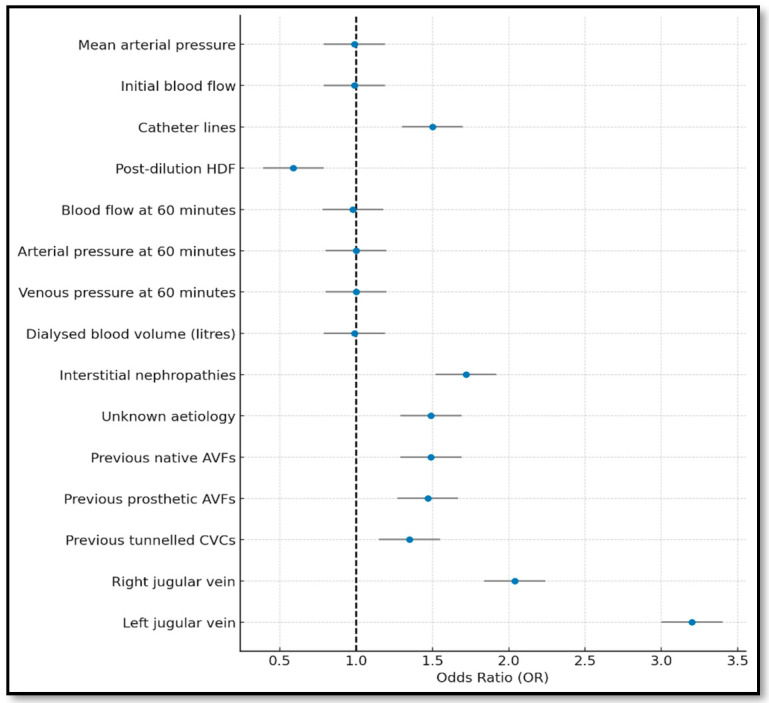
Forest plot of the backward stepwise model (variables with *p*-value < 0.05).

As shown in Figure 2 and Figure 3, both selection approaches identified variables with superior predictive performance. In the forward model, line inversion increased dysfunction risk by 47%, whereas post-dilutional haemodialysis and higher blood flows reduced it. Left jugular vein placement posed the greatest risk [OR = 3.2; 95%IC (2.15–4.78)]. The model fit was acceptable (Hosmer–Lemeshow: *p* = 0.0013; Table 1).

The backward model showed consistent results, again highlighting the negative impact of line inversion, 60 min blood flow, and jugular vein location. Previous vascular access remained protective. This model also demonstrated good fit (Hosmer–Lemeshow: *p* = 0.0094; Table 1).

### 3.3. Lasso Model

**Figure 4:**
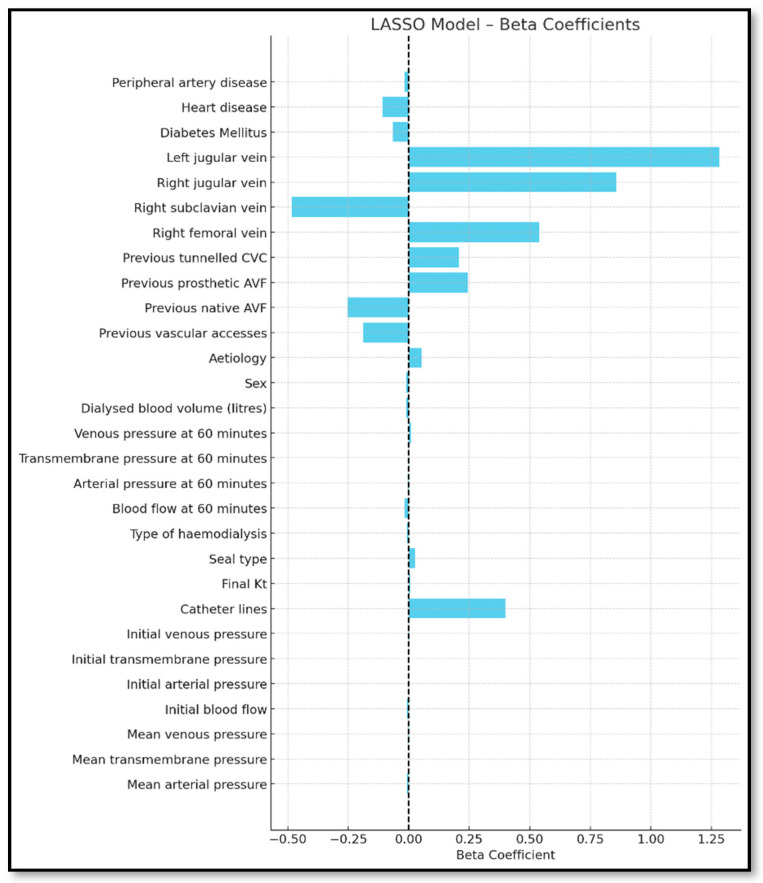
Forest plot of beta coefficients for dysfunction indicators.

The Lasso regression automatically selected the most relevant predictors by eliminating redundancy. A compact set of statistically significant variables was retained, maintaining predictive accuracy. The final set partially overlapped with those in the stepwise models (Figure 4).

### 3.4. Bootstrapping Model

The bootstrapping model was built from 200 randomly generated samples using replacement, followed by variable selection via forward and backward stepwise approaches. This allowed identification of the most consistent predictors. The Hosmer–Lemeshow test yielded a value of 0.0016 (Table 1), indicating excellent agreement between observed and predicted outcomes.

Increased mean arterial pressure and initial blood flow were associated with a 1% decrease in dysfunction risk, both statistically significant (*p* = 0.00).

Inverted lines increased dysfunction risk by 48%, while left jugular vein placement raised event probability by 221%, one of the strongest predictors (*p* = 0.00).

Although vascular disease was linked to an 18% higher dysfunction probability, the association was not statistically significant (*p* = 0.21).

**Figure 5:**
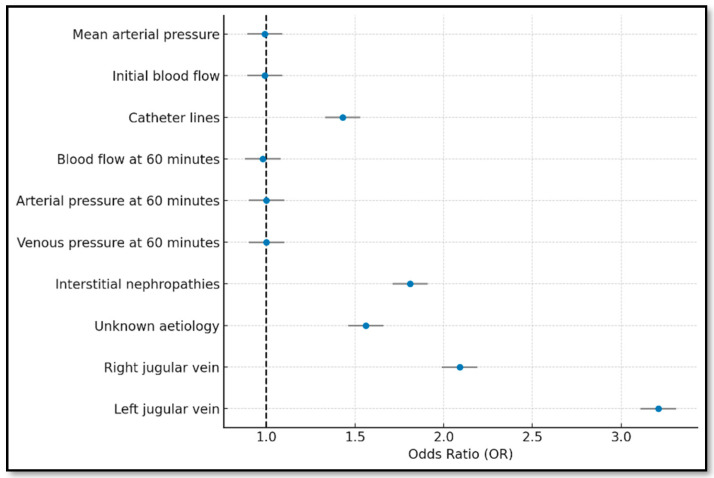
Forest plot of the bootstrapping model (variables with *p*-value < 0.05).

Overall, the bootstrapping model was notable for its robustness, simplicity, and high discrimination, positioning it as a leading predictor of TCVC dysfunction in subsequent HD sessions (Figure 5).

### 3.5. Internal Validation of the Predictive Model

Following the training phase, the forward, backward, and bootstrapping models were chosen for internal validation due to their practicality, simplicity (fewer variables), and favourable fit indicators (lower AIC/BIC and good calibration by the Hosmer–Lemeshow test).

Model discriminative ability was assessed using the area under the ROC curve (AUC). All models exceeded the minimum acceptable threshold (AUC > 0.6), indicating strong performance. The forward and backward model each achieved an AUC of 0.833, while the bootstrapping model yielded 0.832, confirming their clinical value.

In this phase, the discriminative ability of the three preselected models was assessed through internal validation. Validation was conducted on an independent, stratified sample comprising one-third of the original dataset.

Model discrimination, the ability to distinguish sessions with versus without dysfunction, was quantified using the AUC, with ≥0.6 as the minimum threshold for acceptability.

The forward stepwise model achieved an AUC of 0.844, the backward model 0.843, and the bootstrapping model also 0.843, all reflecting robust predictive ability.

Finally, the models were applied to the validation dataset using the logistic regression formula and β coefficients from training. This produced individualised dysfunction probabilities, supporting practical implementation.

### 3.6. Model Selection and Development of the Predictive Tool

The bootstrapping model was ultimately selected based on its ease of use, reduced variable count, and strong statistical performance (i.e., lower AIC/BIC and superior Hosmer–Lemeshow fit; Table 2).

The cut-off point for predicting the event was defined as the one with the highest sensitivity and specificity on the ROC curve of the bootstrapping model, in order to establish an appropriate percentage of discrimination for the occurrence or non-occurrence of TCVC dysfunction (Table 3).

The tool was built in Excel^®^, incorporating the logistic regression equation and the β values of the variables from the bootstrapping model (Table 4).

This process yielded a probability for TCVC dysfunction in the next HD session, which was used as a predictor under a predefined rule requiring it to exceed a cut-off threshold.

This threshold was derived from the ROC curve by optimising sensitivity and specificity, thereby confirming the tool’s predictive value.

## 4. Discussion

Tunnelled central venous catheter (TCVC) dysfunction remains a major complication in haemodialysis (HD) patients, affecting not only the effectiveness of renal replacement therapy but also morbidity, vascular access longevity, and overall quality of life [18]. Although various therapeutic options are available once dysfunction occurs, the evidence suggests that many are inadequate, short-lived, or carry additional risks, particularly related to infection or vascular damage [7,19,20].

In this context, the development of predictive models offers a novel strategy for the primary prevention of catheter dysfunction. The ability to anticipate which patients are at higher risk, based on routinely collected data, enables targeted, personalised, and less invasive clinical interventions, an approach rarely addressed in the existing literature.

The models developed in this study estimate individual dysfunction probability using haemodynamic, clinical, and technical parameters gathered during the HD session. Unlike pharmacological strategies described in the literature [21,22], which are reactive, our model uses predictive logic to promote early therapeutic adjustments, thereby reducing complication risk and avoiding invasive procedures.

## 5. Limitations

### 5.1. Review of Traditional Approaches: Limitations and Opportunities

Conventional measures for managing catheter dysfunction include flushing, thrombolysis with alteplase, anticoagulants, catheter replacement, or radiological procedures such as angioplasty [2,5]. However, these approaches often fail to resolve the underlying pathology. As Szymańska et al. [23], highlight, most dysfunctions stem from fibrin sheath formation, which is difficult to reverse with thrombolytics or irrigation alone. Success rates remain low, particularly in cases of mural thrombosis or central venous stenosis, leading to recurrence and cumulative vascular access deterioration [4,8].

Despite the use of agents such as heparin, citrate, or urokinase for catheter sealing, the risk of haemorrhagic complications persists—especially in frail patients with multiple comorbidities [2,5]. Therefore, continuous vascular access evaluation becomes essential. In this setting, predictive tools can reduce dependence on pharmacological or rescue procedures, promoting a preventive care model.

The reviewed studies focus primarily on pharmacological or mechanical strategies, with little attention paid to predictive surveillance [21,22]. Our model addresses this gap by providing an evidence-based, clinically feasible tool that can be integrated into existing HD protocols.

### 5.2. Predictive Variables: Consistency and Differences with the Literature

The clinical relevance of our model is underscored by the consistency of its predictors with prior studies. For instance, Venegas Justiniano et al. [24] identified advanced age and diabetic nephropathy as dysfunction risk factors, in agreement with our results. Similarly, Chouhaniet al. [25], reported associations between female sex, heart disease, and late-onset TCVC dysfunction, which aligns with our findings.

Catheter location is another well-studied determinant. Wang et al. [26], Fry et al. [27], and Ge et al. [17] all reported increased dysfunction risk with left jugular vein access—likely due to increased intravascular length, tortuosity, and difficulty maintaining optimal tip position. Our results reinforce this observation, showing the highest odds ratios for this site.

Nevertheless, some studies have reported no significant association with sex, age, or anatomical location [24]. Such discrepancies may reflect differences in study design (e.g., longitudinal vs. cross-sectional), population characteristics, or definitions of catheter dysfunction.

Moist [28] and Alonso et al. [29] have linked vascular access characteristics to dialysis adequacy (Kt or Kt/V), emphasising how access type, flow rate, and session duration influence effective dialysis dose. Our findings corroborate this relationship, with effective flow, cleared blood volume, and the use of post-dilutional HDF all significantly associated with dysfunction risk.

Recent studies also highlight the predictive utility of inlet and outlet pressures, line inversion, and sealing type [13,30]. These indicators were incorporated into our final model, reinforcing its scientific validity and clinical relevance.

### 5.3. Clinical Relevance and Model Applicability

The predictive model validated in this study demonstrates high discriminative power (AUC > 0.84 across models) and is built on objective data collected during routine HD sessions. This makes it readily implementable without additional equipment or costs.

Unlike other models—such as García-López’s [31]—which depend on clinical parameters and long-term follow-up, ours uses a cross-sectional and session-based approach, enabling real-time risk identification and early intervention.

The simplicity of the final bootstrapping model and its internal validation further support its use in everyday clinical practice. Beyond prediction, it serves as a decision-support tool that enhances the clinical judgement of the HD team with objective, data-driven insight.

## 6. Conclusions

This study offers a significant contribution to the predictive management of tunnelled central venous catheter (TCVC) dysfunction. The use of a large, real-world dataset, combined with robust multivariable modelling and internal validation, provides strong methodological support and enhances the reliability of the findings. Notably, the final bootstrapping-based model was not only statistically validated but also implemented as a clinically applicable predictive tool through the incorporation of a logistic regression formula and β coefficients, allowing individualised risk calculation in routine practice. An optimal cut-off point was also established to maximise sensitivity and specificity, enabling binary clinical classification. While these results demonstrate the model’s practical value, external validation in diverse populations is recommended to assess its generalisability and long-term performance.

In conclusion, early risk detection using a validated and operational predictive model may improve vascular access surveillance, reduce catheter-related complications, optimise dialysis efficacy, and lower the healthcare costs associated with rescue interventions or catheter replacement. This development represents a meaningful step toward safer, more proactive, and personalised haemodialysis care.

## 7. Patents

**Registered** 16/2025/4094, Copyright Complutense University of Madrid, 2025. All rights reserved.

## Figures and Tables

**Table 1:** Calibration of predictive models.

	Complete	Forward	Backward	Lasso	Bootstrapping
**AIC**	6873.113	7146.702	7144.51	6873.113	7192.647
**BIC**	7206.537	7326.752	7350.278	7206.537	7329.895
**Hosmer-Lemeshow**	0.0231	0.0013	0.0094	0.0231	0.0016

**Table 2:** Area under the ROC curve for the bootstrapping model.

Area Under the Curve	Standard Deviation	*p*-Value	Confidence Interval 95%	
			Upper Limit	Lower Limit
**0.844**	0.01	0.0000	0.82	0.86

**Table 3:** Selected Cut-Off Point from the Area Under the ROC Curve of the Bootstrapping model.

Cut-off Point	Sensitivity	1-Specificity	Specificity
**0.019**	0.816	0.291	0.709

**Table 4:** Internal validation of the bootstrapping model.

Bootstrapping		
Dysfunction Indicator		Beta
**Mean arterial pressure**		−0.007
**Initial blood flow**		−0.006
**Catheter lines**		0.393
**Blood flow at 60 min**		−0.019
**Arterial pressure at 60 min**		−0.004
**Venous pressure at 60 min**		0.005
**Aetiology**	Vascular disease	0.162
	Glomerular disease	0.231
	Congenital and hereditary nephropathies	0.076
	Interstitial nephropathies	0.596
	Prolonged urinary tract obstruction	0.423
	Systemic diseases	0.436
	Unknown aetiology	0.476
**Tunnelled central venous catheter location**	Right jugular vein	0.739
	Left jugular vein	1.167

## Data Availability

The datasets generated and/or analysed during the current study are not publicly available but are available from the corresponding author (V.G.H.) upon reasonable request.
